# Importance of nutritional assessment tools in the critically ill patient: A systematic review

**DOI:** 10.3389/fnut.2022.1073782

**Published:** 2023-01-30

**Authors:** Vicente Domenech-Briz, Vicente Gea-Caballero, Michal Czapla, Elena Chover-Sierra, Raúl Juárez-Vela, Ivan Santolalla Arnedo, Víctor J. Villanueva-Blasco, Juan Luis Sánchez-González, Antonio Martínez-Sabater

**Affiliations:** ^1^Unidad de Epidemiología, Centro de Salud Pública de Xátiva, Valencia, Spain; ^2^Faculty of Health Sciences, Valencian International University, Valencia, Spain; ^3^Research Group Community Health and Care, SALCOM, Valencian International University, Valencia, Spain; ^4^Department of Emergency Medical Service, Wrocław Medical University, Wrocław, Poland; ^5^Nursing Department, Faculty of Health Sciences, University of La Rioja, Research Group GRUPAC, Logroño, Spain; ^6^Facultat d’Infermeria i Podologia, Nursing Department, Nursing Care and Education Research Group (GRIECE), Care Research Group (INCLIVA), Hospital Clínico Universitario de Valencia, Universitat de Valencia, Valencia, Spain; ^7^Center of Biomedical Research – CIBIR, Logroño, Spain; ^8^Research Group on Health and Psycho-Social Adjustment (GI-SAPS), Valencian International University, Valencia, Spain; ^9^Faculty of Nursing and Physiotherapy, University of Salamanca, Salamanca, Spain

**Keywords:** nutritional assessment, nutritional support, nutritional therapy, nutritional risk and screening, care management, SGA, NRS 2002, MNA

## Abstract

**Background:**

Among the risks of the critically ill patient, one of the aspects to be taken into account is the high probability of occurrence of malnutrition risk (40–50%). This process leads to increased morbimortality and worsening. The use of assessment tools allows the individualization of care.

**Objective:**

To analyze the different nutritional assessment tools used during the admission of critically ill patients.

**Methods:**

Systematic review of the scientific literature related to the nutritional assessment of critically ill patients. Between January 2017 and February 2022, articles were rescued from the electronic databases “Pubmed,” “Scopus,” “CINAHL” and “The Cochrane Library”; which will analyze which instruments are used during nutritional assessment in the ICU, as well as their impact on mortality and comorbidity of patients.

**Results:**

The systematic review was made up of 14 scientific articles that met the selection criteria, obtained from seven different countries. The instruments described were: mNUTRIC, NRS 2002, NUTRIC, SGA, MUST and the ASPEN and ASPEN criteria. All the included studies demonstrated beneficial effects after nutritional risk assessment. mNUTRIC was the most widely used assessment instrument, with the best predictive validity for mortality and adverse outcomes.

**Conclusion:**

The use of nutritional assessment tools makes it possible to know the real situation of patients, and by objectifying situations, to allow different interventions to improve the nutritional level of patients. The best effectiveness has been achieved using tools such as mNUTRIC, NRS 2002 and SGA.

## 1. Introduction

In intensive care units (ICU), critically ill patients are at high risk of developing malnutrition, which is associated with worse clinical outcome (1). The nutritional status of critically ill patients deteriorates quite rapidly after admission, as a consequence of severe catabolism caused by stress, proinflammatory cytokines, and hormones, even when patients are well nourished. Ten days after admission, patients may lose 10–25% of their body protein content (exacerbated in those with multiorgan dysfunction syndrome), with losses of up to 10 kg of body weight, depending on the length of stay (2, 3). Critical illness is usually associated with a state of catabolic stress, accompanied by a systemic inflammatory response together with complications related to increased infectious morbidity, multiorgan failure and prolonged hospitalization (4). The scientific literature reports that malnutrition occurs in 40–50% of critically ill patients (with a risk of malnutrition in 35–50% of all patients) (5, 6). The negative effects of malnutrition derive from the correlation between a negative energy balance and an increase in ICU stay (between 5.4 and 6.6 more days of hospitalization), additional days of mechanical ventilation, more frequent infections and higher mortality (data have been found on the threefold relative risk of death among patients with malnutrition, at 1 and 2 years after discharge) (4–9). In addition, a progressive increase in hospitalization costs derived from patient care is suggested, from an average of £5,000 for patients at low risk of malnutrition to an average of over £8,000 for patients at high risk of malnutrition (9–11).

The clinical course of critical illness can be improved by early enteral nutrition (EN), adequate administration of macro- and micronutrients, and strict control of blood glucose. Reductions of up to 35% in the risk of mortality within 30 days of hospital admission have been observed in those patients randomized to early, individualized nutritional therapy (12). Reductions in mortality after nutritional therapy at 90 days (up to 51% of patients), and decreases in the relative risk of overall mortality up to 6 months after discharge (in approximately 27% of hospitalized patients) are also suggested. Reduced readmission rates have been found in patients who received early nutritional support (4, 12, 13).

However, in clinical practice, despite the recommendations of scientific organizations such as the American Society for Parenteral and Enteral Nutrition (ASPEN), nutritional assessment on admission is not a standardized parameter (1). Moreover, tools such as the “Mini Nutritional Assessment” (MNA) are often used, which have not been designed for use in this type of patient, and may therefore lead to underestimation of risk (1, 2). Some useful tools that we can use to perform a nutritional assessment of patients on admission to the ICU are the Subjective Global Assessment (SGA), on the one hand; and, on the other hand, nutritional screening instruments such as the “Nutrition Risk Screening 2002” (NRS 2002), the “Malnutrition Universal Screening Tool” (MUST), the “Nutrition risk in the Critically ill” score (NUTRIC score) or mNUTRIC (modified NUTRIC) (14). Likewise, ASPEN (4) recommends the determination of nutritional risk in all patients admitted to the ICU (1, 2), since from the nutritional assessment it is possible to determine the nutritional diagnosis and establish a correct nutritional intervention (4, 9).

The use of nutritional therapy is aimed at achieving metabolic optimization and attenuation of stress-induced immune responses (derived from critical illness), and not only at avoiding malnutrition (2, 4, 7, 12). Given that, due to their situation, critically ill patients cannot maintain an adequate intake, nutritional therapy is part of the treatment, with early EN being indicated in patients with a functional gastrointestinal tract and hemodynamic stability (4, 7, 13). Thus, in recent years, there has been a transition from the concept of nutritional support to that of nutritional therapy, as the benefits of early administration of EN (before 24–72 h) have been demonstrated in the metabolic response to stress, prevention of oxidative cellular injury and improved immune response (4, 7, 12, 13, 15).

In order to establish adequate and individualized guidelines, it is necessary to carry out an individualized nutritional evaluation in the first hours after admission to hospital units, and mainly in critical care units (4, 9, 14), allowing the detection of the risk of malnutrition, and the early initiation of an adequate nutritional therapy for each person that allows minimizing the adverse effects (9, 13).

This nutritional assessment will include information regarding dietary history; nutrient intake; anthropometric and biochemical measurements; physical, clinical and disease conditions; and functional status (4, 9, 13), and allows the adequacy of supportive therapy to organic functions (4, 13, 15). Thus, the research question that emerges from this systematic review is: What are the benefits of using nutritional status assessment on admission in critically ill patients, and which tools is most effective?

The objective of our study is to identify and describe the tools most commonly used in nutritional assessment in critical care units, and to determine how nutritional assessment and therapy are able to reduce malnutrition and morbidity and mortality in critically ill patients (Table 1).

**TABLE 1:** PICO format question.

Research question PICO format	
Patient	Patients admitted to the ICU
Intervention	Nutritional screening and assessment
Comparation	Comparison of nutritional assessment scales
Outcomes	The effect of nutritional assessment on patients’ health status

## 2. Methodology

### 2.1. Study design

Systematic review of the scientific literature conducted in the year 2022, using the PRISMA (Preferred Reporting Items for Systematic reviews and Meta-Analyses) 2020 statement (16). The review protocol was registered in the Prospective International Registry of Systematic Reviews (PROSPERO), with registration number CRD4202222328783.

### 2.2. Search strategy

The data retrieved for the review was from the last 5 years (01/01/2017 and 01/02/2022). A search was performed in the following electronic databases: “Pubmed,” “Scopus,” “CINAHL” and “The Cochrane Library.” The free and “Mesh” terms used were: “nutrition assessment,” “nutritional support,” “nutrition therapy,” “nutritional risk and screening,” “care management,” “critical care,” “adult.” The search was limited to articles found in English, Spanish or Portuguese. The bibliographic references of the retrieved articles were examined with the aim of finding other relevant articles (reverse search). The selected articles were grouped according to the type of study and study variables (most commonly used tools; presence of malnutrition, inflammation or morbimortality analysis in critically ill patients) in order to be able to establish and evaluate the evidence. The bibliographic manager “Mendeley Reference Manager” was used to manage the retrieved documents.

The following table (Table 2) shows the search strategy used to retrieve the eligible documents in this systematic review, as well as the terms used in each database, the search period selected and the articles obtained.

**TABLE 2:** The search strategy.

Database	Search strings	Articles retrieved	Articles selected
Pubmed	(Nutritional assessment) AND (intensive care unit) AND (critical illness) NOT (pediatrics)	71	7
	(((Nutritional risk screening and assessment) AND (intensive care unit) AND (critical illness)) NOT (pediatrics))	60	
	(((Nutrition assessment) AND (intensive care units)) AND (care management)) AND (critical illness)	84	
	((((nutritional assessment) AND (nutritional support)) AND (intensive care units)) AND (critical illness)) AND (tool)	23	
	(((Nutritional assessment) AND (nutritional support)) AND (intensive care units)) AND (nurse)	12	
	((Nutrition assessment) AND (intensive care units)) AND (critical illness)	226	
	((((((Nutrition assessment) AND (intensive care units)) AND (care management)) AND (critical illness)))) AND (nursing care)	13	
Scopus	(Nutritional assessment) AND (intensive care unit) AND (critical illness) NOT (pediatrics)	63	0
Cinahl	(Nutritional assessment) AND (nutritional support) AND (intensive care unit) NOT (pediatrics)	32	3
	(Nutritional assessment) AND (intensive care unit) NOT (pediatrics)	32	
	(Nutritional risk screening and assessment tools) AND (intensive care unit)	11	
Cochrane Library	(Nutritional assessment) AND (nutritional risk and screening) AND (intensive care units) NOT (pediatrics)	15	2
	(Nutritional risk and screening) AND (intensive care units) NOT (pediatrics)	17	

### 2.3. Selection criteria

Inclusion criteria: Studies addressing the importance of nutritional screening and assessment on admission of critically ill patients in intensive care units. Evaluation of the predictive capacity of adverse outcomes (malnutrition or inflammation) and mortality. Patients evaluated who are older than 18 years of age. Types of studies: systematic reviews, randomized controlled trials, observational studies and cross-sectional studies (16, 17).

Exclusion criteria: studies on pediatric patients or those belonging to other hospitalization units. Studies focused on pharmaceutical properties of EN or PN or those in which the performance of nutritional risk and complete nutritional assessment is not evaluated. The following types of publication: editorials, letters, legal cases, interviews, book chapters, commentary articles, news, review studies, methodological considerations. Research that is not conducted for humans. Duplicate studies.

### 2.4. Effect measures

The evaluation of methodological quality was carried out in two phases: first, the evaluation/critical reading of each document and, subsequently, verification of the level of bias. For the quality assessment, the scale adjusted to the corresponding design was used: PRISMA (16), STROBE (“Strengthening the reporting of observational studies in epidemiology”) (18) or CASPe (“Critical Appraisal Skills Programme”) critical reading (19). As for the assessment of risk of bias, the NOS (“Newcastle-Ottawa”) scale was used for longitudinal non-randomized studies (20, 21), the ROB (“Risk-of-bias tool”) scale for randomized clinical trials (22) and the ROBIS (“Risk of Bias in Systematic Reviews”) scale for systematic reviews (23, 24). The latter two are two instruments recommended by the Cochrane Collaboration (22, 24). For the studies evaluated using the NOS scale, those with scores of less than seven points were defined as having a high level of bias (25, 26).

Finally, the Scottish Intercollegiate Guidelines Network (SIGN) tool (27) was used to evaluate and classify the studies according to the level of evidence.

### 2.5. Data extraction (selection and codification)

The selection of documents was done first by title and secondly by reading the abstract. The selection was made by two independent investigators to identify studies that potentially met the inclusion criteria described above. For potentially eligible studies, the full text was retrieved and also evaluated by both reviewers for eligibility. A third investigator served as a reviewer in the case of discrepancy between the two. For each study, data were recorded on a form, including the study characteristics (population, study design) and the primary topic (nutritional assessment methods, whether screening or full assessment tools).

### 2.6. Data summarization strategy

A narrative synthesis of the findings of the included studies was made, structured according to the type of intervention, the content of the same, the results and the characteristics of the target population.

## 3. Results

**FIGURE 1:**
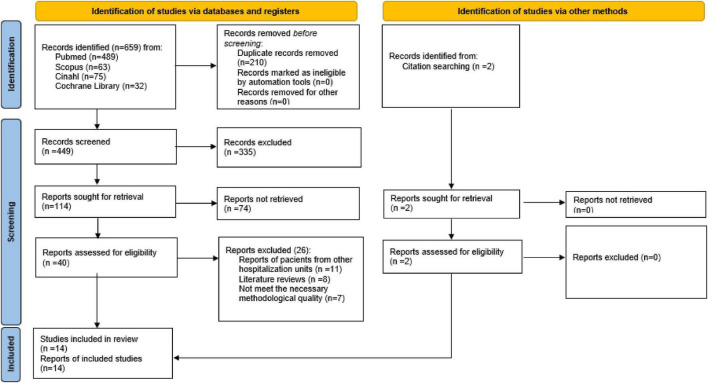
Selection process flow chart (PRISMA 2020).

The first search showed a total of 659 articles, of which 12 were finally selected, in addition to 2 articles found by means of a reverse search, so that 14 articles were finally obtained for the systematic review. The selection process is shown in Figure 1.

As for the design of the studies, a systematic review (26), a randomized controlled trial (28), 6 retrospective longitudinal studies (3, 29–33), 5 prospective longitudinal studies (34–38) and 1 cross-sectional study (39) were collected. And by provenance, 4 were from China (28, 31, 35, 36), 4 from Brazil (3, 26, 37, 38), 2 from Iran (32, 39), 1 from Israel (29), 1 from the United States (30), 1 from Australia (34) and 1 from Greece (33).

### 3.1. Evaluation of the level of bias

All the studies included in the present systematic review were rated with a low level of bias (3, 26, 28–33, 35–39) except the one by Egan et al. (34), with a score of 6 on the NOS scale. The longitudinal studies presented a mean of 7.58 points on the NOS scale (21). For the systematic review of Cattani et al. (26) the “Robis” tool (23, 24) was used, with a “low risk of bias” result. In the randomized clinical trial of Liu et al. (28) the “RoB” scale (22) was used, with the same result: “low risk of bias.”

### 3.2. Instruments and criteria used

**FIGURE 2:**
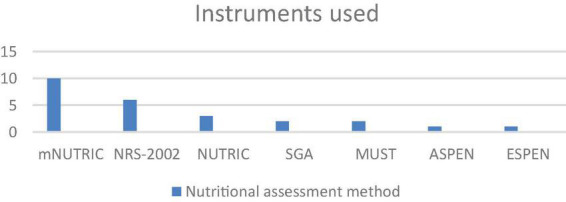
Method to assess nutritional status.

The most commonly used nutritional assessment tool was mNUTRIC (3, 26, 30–33, 35–39), followed by NRS 2002 (3, 26, 28, 29, 36, 37), NUTRIC (26, 29, 32), SGA (29, 38), MUST (26, 34) and the ASPEN and ESPEN criteria (29) (Figure 2).

The mNUTRIC score was scored in all the articles found (3, 26, 29–39) using 5 variables: age, APACHE II score, SOFA score, number of comorbidities, and days since ICU admission. Most authors (3, 30, 31, 33, 35–37, 39) determined that this tool was easier to complete than the original NUTRIC tool, due to the absence of the variable IL-6 (Interleukin-6), which was more difficult to obtain and not all ICUs analyzed had access to this laboratory parameter.

NRS 2002 was the second most used tool (3, 26, 28, 29, 36, 37), where uniformity in its application was also found. First, an initial screening was carried out, taking into account BMI, weight loss, reduction of intake in the last week and severity of the disease. Subsequently, nutritional status and disease severity were assessed more specifically. However, the determination of nutritional risk varied between studies, where some established patients at nutritional risk with a score ≥3 (28, 30, 36), and others with a score ≥5 (3, 29).

NUTRIC was the third most employed tool (26, 29, 32). Age, APACHE II score, SOFA score, number of comorbidities, days since admission to the ICU and the IL-6 parameter. This assessment tool was less employed than its modified version due to IL-6, as it was a difficult value to obtain.

The fourth nutritional assessment tool was the SGA (29, 38). The SGA consisted of a questionnaire that included nutritional history (weight loss, dietary changes, gastrointestinal symptoms), physical examinations performed 24 h post-admission (degree of muscle loss, subcutaneous fat loss or presence of edema) and the impact of the disease.

MUST was also described by two articles (26, 34). MUST is a 5-step tool that incorporates BMI, weight loss and the effect of acute illness. In this case, the synthesis of the studies offered by Cattani et al. (26) and the prospective study by Egan et al. (34) did find similarities in terms of application and determination of nutritional risk.

Finally, the ASPEN and ESPEN criteria were only described in one article (29), which take into account etiological and phenotypic characteristics of the patients. These criteria are the ones taken into account to determine the diagnosis of malnutrition by these nutrition societies.

The characteristics of the tools were summarized in Table 3, showing which parameters are common to the nutritional assessment instruments described above.

**TABLE 3:** Components of the different screening tools.

Features	mNUTRIC	NRS 2002	NUTRIC	SGA	MUST	ASPEN	ESPEN
Age	X	X	X				
Apache II	X		X				
SOFA	X		X				
Comorbidities	X		X				
Days of hospital admission	X		X				
IL-6			X				
IMC		X		X	X	X	X
Percentage of weight loss		X			X	X	X
Energy intake compared to energy needs		X				X	X
Severity of illness		X		X	X	X	X
Ener				X	X		
Muscle loss						X	X
Metabolic stress				X			
Physical examination				X			
Nutritional risk classification	<to 3: low risk ≥to 4: high risk ≥5: high risk	<to 3: low risk ≥3: risk ≥to 5: high risk	≤to 5: low risk ≥to 6: high risk	A: well nourished B: moderately malnourished C: severely malnourished	0: low risk 1: medium risk ≥to 2: high risk	Phenotypic criteria: unintentional weight loss, low BMI or loss of muscle mass Etiological criteria: decreased intake or presence of morbidity At least one etiologic criterion and one phenotypic criterion	
Number of studies that have described this tool	11	6	3	2	2	1	1

### 3.3. Effects of nutritional assessment

All the included studies (3, 26, 28–39) demonstrated beneficial effects after nutritional risk assessment in critical patients: improving patient prognosis when receiving individualized nutritional therapy (28, 31, 38), identifying patients at nutritional risk with a higher probability of morbidity and mortality who could benefit from nutritional support (26, 29, 32, 33, 35–38) or improving the adequacy of the energy needs of patients admitted to the ICU (30, 39). Correct nutritional screening and assessment allowed the identification of patients who could best benefit from individualized nutritional therapy, as could be seen in the RCT of Liu et al. (28), where patients who received individualized nutritional therapy had a higher rate of improvement in the experimental group (65.1 vs. 45.1%) and the mortality rate was lower than that of the control group (2.3 vs. 6.1%).

These data also correlated with the other results found (26, 31, 35–38), revealing that there was a higher mortality in the groups classified as high nutritional risk by mNUTRIC, NRS 2002 or SGA; so that early nutritional therapy had to be established in these patients to protect them from the risk of malnutrition. The variables most commonly used to determine the benefit of using nutritional assessment tools were mortality (26, 28, 29, 31, 33, 35–38), the presence of comorbidity or complications (3, 26, 28, 31, 32), increased hospital stay or readmissions (31, 35, 37) and the adequacy of energy requirements (26, 30, 39). The analyses for the calculation of mortality risk differed according to the types of studies and the tools used:

• Risk of 28-day mortality for patients at high nutritional risk: 87% mortality in the case of Zhang et al. (34) and 67.4% mortality in the study by Wang et al. (35), using mNUTRIC.

• Significant increase in the 28-day mortality rate among patients classified as high nutritional risk using mNUTRIC and NRS 2002 (36). Machado et al. (37) found that patients at high nutritional risk according to mNUTRIC had a threefold increased risk of in-hospital mortality, whereas patients considered at high nutritional risk according to NRS 2002 did not have a statistically higher increased risk of death. The use of the mNUTRIC tool by Gonzalez et al. (38) concluded similar results, detecting a 2.37 and 2.97 times higher mortality risk (depending on the cut-off point used) in patients classified as nutritional risk (score ≥ a 5 or ≥ a 6); whereas patients classified at risk according to score of ≥4 had an almost 6 times higher mortality risk after 28 days than individuals classified without nutritional risk.

• Use of two tools for nutritional assessment: Machado et al. (37) and González et al. (38) proposed the use of mNUTRIC combined with another nutritional assessment tool: NRS 2002 in the first case (37) and SGA in the second (38). Gonzalez et al. (38) suggested that one death could be avoided for every 1.62 patients identified as being at nutritional risk by mNUTRIC and with severe malnutrition (SGA “C”) who received an individualized nutritional intervention.

The data shared reveal that the use of any nutritional assessment tool on admission of critically ill patients is effective in detecting the risk of mortality. In addition, other results described were the relationship between nutritional risk and increased risk of presenting comorbidities or longer stay in the ICU (28, 31, 32, 36).

Another way of detecting the positive effects of the use of nutritional assessment instruments could be observed in other studies (30, 39), since the mNUTRIC instrument was proposed to predict energy, protein, carbohydrate and fat intake; because mNUTRIC scores were strongly associated with calorie and protein requirements.

A summary of all the selected papers can be found in the Summary Table (Table 4).

**TABLE 4:** Summary table.

References	Design sample	Intervention tool used	Variables results	Results	Conclusions	Evaluation of the study report/Risk of bias	Level of evidence: SIGN
Egan et al. (34). Australia.	Prospective observational study –20 patients admitted to the ICU on non-invasive mechanical ventilation.	–To compare the time required of patients in whom nutritional screening was completed, using both MUST and mNUTRIC tools in critically ill patients. / -MUST and mNUTRIC.	-Time taken (minutes) to complete nutritional risk screening using both tools. -Barriers found in the use of the nutritional screening tools	-Screening using the MUST tool took less time to complete than screening with mNUTRIC. -The maximum time spent with MUST was 14 minutes, compared with 33 minutes for mNUTRIC.	-MUST is the most feasible nutritional risk screening tool for use in a cohort of ICU patients on non-invasive mechanical ventilation, as it required less time and fewer barriers to completion, as opposed to mNUTRIC.	STROBE: 20/22 / NOS: 6/9	2–
Zhang et al. (36). China.	Prospective observational study. –140 patients admitted to the neurological ICU.	-Investigate the NRS 2002 and mNUTRIC nutritional screening tools in the setting of critically ill neurological patients to predict the prognosis of these patients. / -NRS 2002 and mNUTRIC	-Prevalence of nutritional risk -Mortality of patients at 28 days.	-High nutritional risk as determined by NRS 2002 and mNUTRIC was associated with a significantly increased 28-day mortality rate. -Compared between groups of patients in whom mNUTRIC had been used, those at high nutritional risk according to this tool showed a significantly higher incidence of pulmonary infection, hospital infection, organ dysfunction, and increased 28-day mortality rate, as opposed to those diagnosed at low nutritional risk.	-The mNUTRIC score is independently related to the risk of 28-day mortality in critically ill neurological patients.	STROBE: 22/22 / NOS: 8/9	2 ++
Javid et al. (39). Irán.	Cross-sectional observational study. –1321 patients admitted around 50 ICU’s of 25 hospitals in Iran.	-To assess the nutritional adequacy of patients considering the diagnosis and prevalence of malnutrition on admission. / -mNUTRIC	–Nutritional intake. -Classification of patients according to mNUTRIC.	–The mean calorie and protein adequacy was 16.2% and 10.7%, respectively. 16.2% and 10.7%, respectively. -Patients classified as high nutritional risk had a higher adequacy index than those at low nutritional risk.	-The nutritional intake of patients admitted to the ICU was very low. -Calorie and protein requirements were underestimated. -The mNUTRIC score can predict the energy intake of critically ill patients. -It is recommended to perform a complete nutritional assessment on the first day of hospitalization in order to correctly estimate energy needs.	STROBE: 22/22 / NOS: 8/9	2 +
Zhang et al. (31). China.	Retrospective observational study. –136 patients admitted to different ICU’s in China.	To investigate the applicability of mNUTRIC score to assess nutritional risks and predict outcomes of critically ill COVID-19 patients. / -mNUTRIC	–Medical data, mortality and complications of patients. -mNUTRIC score of nutritional risk.	-A large proportion of critically ill COVID-19 patients were at high nutritional risk on admission to the ICU. -Patients with high nutritional risk on ICU admission had significantly higher 28-day mortality in the ICU, as well as twice the likelihood of ICU death at 28 days (compared with those identified as having low nutritional risk).	-The mNUTRIC score may be a suitable tool for nutritional risk assessment and prognostic prediction in critically ill COVID-19 patients.	STROBE: 22/22 / NOS: 8/9	2 ++
Wang et al. (35). China.	Prospective observational study. –3107 critically ill patients admitted to the ICU.	-To identify nutritional risk in patients admitted to the ICU using the mNUTRIC score; and to describe the relationship between 28-day mortality and elevated nutritional screening scores. / -mNUTRIC	-mNUTRIC score. -Health-related variables (age, BMI, drug use, etc.) and mortality data. -Length of stay of patients in the ICU.	-Mortality at 28 days for the maximum mNUTRIC score was 67.4%. -The mNUTRIC score was an independent predictor of 28-day mortality, which increased by 8.5% for each point on the nutritional screening tool. -Higher mNUTRIC scores were associated with longer ICU stay.	-The mNUTRIC tool is a good tool for nutritional risk assessment in critically ill patients; it is practical and easy to use.	STROBE: 22/22 / NOS: 8/9	2 ++
Machado et al. (37). Brasil.	Prospective cohort study. –384 patients admitted to the ICU.	-To evaluate the performance of mNUTRIC, independently or combined with NRS 2002, in predicting hospital mortality in critically ill patients admitted to the ICU. / -mNUTRIC y NRS 2002.	-Nutritional screening -Length of hospital and ICU stay, in-hospital death, ICU readmission.	-Patients classified as nutritional risk according to mNUTRIC had a 3-fold higher risk of in-hospital mortality. -Patients classified as high nutritional risk according to NRS 2002 did not have a statistically significant increased risk of death. -Patients classified as nutritional risk by both tools had a 2-fold increased risk of in-hospital mortality.	-The NRS 2002 and mNUTRIC nutritional screening tools performed well as predictors of mortality, alone or in combination. -The mNUTRIC had better discriminative ability to quantify the risk of hospital mortality in critically ill patients.	STROBE: 20/22 / NOS: 9/9	2 +
Coruja et al. (3). Brasil.	Retrospective observational study. –208 patients admitted to the ICU.	- To compare the detection of nutritional risk by mNUTRIC and NRS 2002, to identify if they are concordant tools. -NRS 2002 y mNUTRIC	- mNUTRIC and NRS 2002 scores during the first 24 h after admission of patients to the ICU. -Prevalence of nutritional risk determined by screening tools.	-The most frequent component of the NRS 2002 was the severity of illness score. -The component most evaluated by mNUTRIC was the number of comorbidities. -There was fair concordance between the two nutritional risk screening tools.	- NRS 2002 and mNUTRIC identify nutritional risk differently, so the two instruments are proposed as not interchangeable.	STROBE: 21/22 / NOS: 7/9	2 +
Rattanachaiwong et al. (29). Israel.	Retrospective observational study. –120 patients admitted during the study period.	-To apply different nutritional assessment and screening tools (ASPEN and ESPEN criteria, NRS 2002 and mNUTRIC). -To compare these classifications with the SGA.	-Nutritional status. -Concordance of the different tools. -Mortality.	-NRS 2002 showed the highest sensitivity for identifying severe malnutrition. -NRS 2002, ASPEN and ESPEN criteria showed the highest specificity with GHS. -mNUTRIC had lower performance in detecting malnutrition.	-None of the tools showed an association with mortality after adjustment.	STROBE: 18/22 / NOS: 8/9	2 +
Cattani et al. (26). Brasil.	Systematic review. –36 selected articles that met the inclusion criteria.	-Summarize the evidence regarding the prevalence of nutritional risk and the predictive validity of the different nutritional risk screening instruments used in critically ill patients. / -Different screening tools found: mNUTRIC, NRS 2002, MUST, Nutritional Score Risk (NSR)	-Prevalence of nutritional risk -Predictive validity of nutritional screening tools.	-The mean prevalence of nutritional risk in critically ill patients was 55.9%. -The most commonly used instruments were the mNUTRIC and the NRS 2002. -Nutritional risk was an independent predictor of 28-day mortality.	- The prevalence of nutritional risk in critically ill patients varies widely. -Identification of patients at nutritional risk is not a simple task, but it is clinically relevant. -NRS 2002 and mNUTRIC could be the current tools available for nutritional risk assessment, due to their proven validity.	PRISMA: 26/27 / ROBIS: Low risk of bias.	1–
Liu et al. (28). China.	Randomized controlled trial. –220 patients admitted to the neurological ICU of a hospital in China.	-Individualized nutritional risk assessment and screening of the experimental group, with treatment prescription and review of the effects on the patients. / -NRS	-Incidence of pulmonary infection, hypoproteinemia, mechanical ventilation, hospitalization time, improvement and mortality rate.	-Nutritional assessment was able to diagnose malnutrition and establish correct nutritional support. -The number of patients at nutritional risk after therapy was reduced in the experimental group. -In the experimental group, the incidence of hypoproteinemia and pulmonary infection was reduced, hospitalization days were decreased, the rate of improvement of patients was increased and the mortality rate was decreased.	-Systematic nutritional assessment provided a theoretical basis for nutritional support in neurocritical patients. -The prognosis of patients who received individualized nutritional therapy was better.	CASPe: 9/11 / RoB: Low risk of bias.	1 +
Canales et al. (30). Estados Unidos.	Retrospective observational study. –312 patients admitted to the ICU.	-Compare mNUTRIC with NRS 2002 in terms of its relationship with macronutrient deficits in critically ill patients. / -mNUTRIC and NRS 2002	-Protein-calorie deficit. -Association with nutritional screening instruments.	-mNUTRIC scores are strongly associated with protein and calorie requirement; whereas no relationship was found between these requirements and NRS 2002.	-Larger studies are needed to validate the findings. -mNUTRIC is more closely matched to energy requirements than NRS 2002. -The use of these tools could improve clinical outcomes in patients at nutritional risk.	STROBE: 22/22 / NOS: 8/9	2 +
Gonzalez et al. (38). Brasil.	Prospective longitudinal observational study. –205 patients hospitalized in the ICU.	To compare the prognostic power of mNUTRIC and SGA, independently or simultaneously, to predict the risk of 28-day mortality following admission of critically ill patients. / -mNUTRIC and SGA.	-Nutritional screening by SGA and mNUTRIC (cut-off points). -Mortality risk -Number needed for screening (NNS).	-Patients classified as nutritional risk by mNUTRIC and as severely malnourished by SGA (SGA “C”), showed a risk of death after 28 days of ICU admission was more than 7 times higher, compared to patients without nutritional risk by mNUTRIC (regardless of nutritional status determined by SGA). -According to the NNS, one death could be averted for every 1.62 patients identified as being at nutritional risk by mNUTRIC score and classified as SGA “C” (severely malnourished).	-It is suggested that mNUTRIC be used in the first 24 h of ICU admission to detect patients at increased risk of mortality. -Subsequent nutritional assessment by SGA in patients classified as being at nutritional risk is associated with better identification of patients at increased risk of mortality and those in whom more aggressive nutritional therapy is recommended.	STROBE: 20/22 / NOS: 7/9	2 ++
Eslamian et al. (32). Irán.	Retrospective observational study. –150 patients hospitalized.	-To evaluate the association between intestinal permeability and nutritional status in a group of critically ill patients. / -NUTRIC and mNUTRIC.	-Intestinal markers related to intestinal permeability (zonulin and endotoxin). -Plasma glutamine levels. -NUTRIC and mNUTRIC scores.	–54% of patients were classified as high nutritional risk using mNUTRIC, while the proportion was 47% when NUTRIC was used. -Multivariate analyses showed significant associations between increased zonulin and endotoxin levels and increased NUTRIC or mNUTRIC category.	-Gut permeability-related levels are significantly positively associated with the nutritional risk tools used. -Physicians should evaluate critically ill patients with the NUTIC tool to assess nutritional risk, which may be associated with intestinal permeability.	STROBE: 19/22 / NOS: 7/9	2 +
Chourdakis et al. (33). Grecia.	Retrospective longitudinal observational study. –80 patients admitted to the ICU.	To translate and adapt the mNUTRIC score to the Greek language. To evaluate the predictive ability of mortality. / mNUTRIC.	–mNUTRIC score. -Prevalence of nutritional risk. -Mortality.	-The mNUTRIC tool was considered easy to use, fast and complete. -Cronbach’s alpha was 0.58. -Increased mortality and comorbidities were observed among patients classified as high nutritional risk by mNUTRIC, compared with those at low nutritional risk.	-The Greek version of mNUTRIC was a validated, quick and easy to use tool; which is able to discriminate critically ill patients from benefiting from improved nutrition.	STROBE: 20/22 / NOS: 7/9	2 ++

## 4. Discussion

In the present systematic review we found 14 scientific articles (3, 26, 28–39) describing the benefits of using a nutritional assessment tool (mNUTRIC, NRS 2002, NUTRIC, SGA, MUST and ASPEN and ESPEN criteria): prediction of mortality risk for earlier initiation of nutritional therapy (3, 26, 28, 31, 33–38), reduction in the number of complications and length of stay related to malnutrition (3, 26, 28, 32, 36, 38) or improved adequacy of energy requirements (30, 39).

The strengths of this systematic review have been the inclusion of an exhaustive bibliographic search in 4 large electronic databases: “Pubmed,” “Scopus,” “The Cochrane Library” and “CINAHL” (3 general databases and a specific nursing database), together with the evaluation of the risk of bias of the studies, which has allowed the selection of those with the lowest risk of systematic error. The results found are in agreement with the available scientific evidence (4, 8, 13, 14, 26, 40) showing how critically ill patients can benefit from the use of nutritional assessment tools to improve health and mitigate adverse outcomes. Thus, we can classify these tools as: nutritional risk screening tools (mNUTRIC, NRS 2002, NUTRIC, and MUST) and comprehensive nutritional assessment tools (SGA and the ASPEN and ESPEN criteria).

Most of the longitudinal studies determined the predictive validity of the nutritional screening instruments used (30–32, 35–39), the most analyzed tool with the best predictive capacity for mortality and adverse outcomes being mNUTRIC (3, 26, 30–33, 35–37, 39), as we observed in other scientific studies (41, 42), which suggest that mNUTRIC is a good predictor of mortality in critically ill patients, and that these patients can improve their health status if they are evaluated and given nutritional therapy. However, we detected some unfavorable results after the use of this instrument (29, 34), such as that this tool took longer to complete than others, such as MUST (34). After analysis of these studies, we concluded that they were not too reliable, as they didn’t find any tool that was associated with mortality after adjustment for variables or had a high level of bias (29, 34).

Another critique found for the mNUTRIC score was that no nutritional parameters were explicitly taken into account (26). However, the scientific literature (4, 30–33, 35–39, 41–50) gives value to this tool for the following reasons: It has been validated in the critical patient population based on the malnutrition criteria offered by ASPEN (40); it does not contain classical nutritional variables (weight evolution or recent food intake) due to the difficulty of extracting them in ICU patients; the variables used correlate correctly with the pathophysiology of malnutrition, since the degree of inflammation is a determinant factor of nutritional risk, therefore using APACHE II and the SOFA scale is more convenient; the variables related to the number of comorbidities (they consider chronic inflammation) and days of hospitalization in the ICU (they determine reduced intake) are more objective; it has demonstrated predictive validity for mortality, adverse clinical outcomes and increased length of stay of patients; and finally, it is an easy to apply and low cost tool (after elimination of the IL-6 parameter).

Regarding the mNUTRIC cut-off points, most of the scientific literature classifies nutritional risk as a score greater than or equal to 5 (3, 26, 30, 31, 41–44). However, Gonzalez et al. (38) and Wang et al. (35) found that patients classified as nutritional risk with a score greater than or equal to 4 had a higher risk of mortality than those with a score greater than or equal to 5. If nutritional risk could not be determined by mNUTRIC, our results suggest that another tool used to assess the prognosis of critically ill patients is NRS 2002 (3, 26, 28, 37).

Regarding nutritional assessment tools, which according to ASPEN and ESPEN (4, 13, 14) should be performed in all patients at high risk of malnutrition, the only instrument selected in this review was the SGA (29, 38). In the prospective study by Gonzalez et al. (38) we can see how up to 1 death could be avoided for every 1.62 patients identified as being at nutritional risk according to mNUTRIC and classified as SGA “C” (severe malnutrition). These data are in line with the available evidence (8, 13, 42), which shows how SGA has greater predictive validity than other tools (especially for hospital mortality, length of stay, and complications), such as MNA. In addition, the latest ASPEN, ESPEN and Global Clinical Nutrition Community (GLIM) guidelines (40) determine that at least one phenotypic criterion and one etiological criterion must be available to make a diagnosis of malnutrition; thus, the parameters assessed by GHS can contribute to the development of this diagnosis.

In summary, the results found in this review can benefit the professional practice of nurses and patient outcomes, as they show how nurses are in charge of collecting information and determining nutritional risk using the screening tools analyzed (28, 31, 34, 36, 39). As we know, these tools are key for the prediction of mortality risk, complications or individual protein-energy adequacy. The ability to generate beneficial effects in patients has an impact on improving effectiveness and efficiency, since these tools can save costs and improve patient health outcomes (26, 42).

This study has some limitations. We are aware that observational studies may have more types of biases, such as the risk of selective reporting of the analysis and outcome, being one of the limitations of this study. In our review, most of the studies were not RCTs, and therefore it is recommended that studies with more robust designs [such as RCTs] be conducted to test the true scope of nutritional assessment tools in the health of critically ill patients. Another limitation of this study is the heterogeneity of the instruments found to screen for nutritional risk, since we have found various nutritional risk tools, and the possibility of using them or not depending on the context of the ICU of each hospital.

## 5. Conclusion

The nutritional assessment tools described were mNUTRIC, NRS 2002, NUTRIC, SGA, MUST and the ASPEN and ESPEN criteria. Among these tools, the most widely used and effective were mNUTRIC, NRS-2022 and SGA, either independently or in combination with each other.

The most highly rated tool with the best mortality prediction capacity was mNUTRIC. It was also the most useful for predicting the energy requirements of the patients, so that nutritional therapy could be established in those patients classified as high risk nutritional, with the aim of reducing comorbidity derived from malnutrition and reducing the length of stay of critical patients. Thus, among the tools for assessing nutritional risk, mNUTRIC was the most effective. SGA is a nutritional assessment tool that can complement and support the risk assessment performed by mNUTRIC.

Nutritional risk assessment and screening have been shown to be able to improve malnutrition and health status in critically ill patients. The use of any nutritional assessment tool on admission of critically ill patients is able to detect the risk of mortality, thus allowing earlier initiation of nutritional therapy to improve the prognosis of patients classified as high risk.

## Data availability statement

The original contributions presented in this study are included in the article/supplementary material, further inquiries can be directed to the corresponding author.

## Author contributions

VD-B, VG-C, EC-S, and RJ-V: conceptualization and software. VD-B, AM-S, VV-B, JS-G, and RJ-V: methodology. VD-B, VG-C, RJ-V, MC, and IS: validation. VD-B, VG-C, EC-S, and RJ-V: formal analysis. VD-B, VG-C, MC, and RJ-V: investigation. VD-B, AM-S, VV-B, JS-G, RJ-V, and MC: data curation and writing—original draft preparation. VD-B, VG-C, RJ-V, MC, and IS: writing—review and editing. VD-B: visualization and project administration. RJ-V and AM-S: supervision and funding acquisition. All authors contributed to the article and approved the submitted version.
